# Bypassing the bypass: complex laparoscopic reparation of internal hernia after laparotomic Roux-en-Y gastric bypass

**DOI:** 10.1093/jscr/rjag725

**Published:** 2026-08-23

**Authors:** Giacomo Piatto, Matteo Zuin, Rubina Palumbo, Ylenia Camilla Spolverato, Alfonso Giovanni Recordare

**Affiliations:** U.O.C. Chirurgia Generale, Ospedale di Cittadella (Via Pilastroni, 15, 35013 Cittadella PD)—ULSS 6 Euganea, Cittadella (PD), Italy; U.O.C. Chirurgia Generale, Ospedale di Cittadella (Via Pilastroni, 15, 35013 Cittadella PD)—ULSS 6 Euganea, Cittadella (PD), Italy; U.O.C. Chirurgia Generale, Ospedale di Cittadella (Via Pilastroni, 15, 35013 Cittadella PD)—ULSS 6 Euganea, Cittadella (PD), Italy; U.O.C. Chirurgia Generale, Ospedale di Cittadella (Via Pilastroni, 15, 35013 Cittadella PD)—ULSS 6 Euganea, Cittadella (PD), Italy; U.O.C. Chirurgia Generale, Ospedale di Cittadella (Via Pilastroni, 15, 35013 Cittadella PD)—ULSS 6 Euganea, Cittadella (PD), Italy; Director of U.O.C. Chirurgia Generale, Ospedale di Cittadella—ULSS 6 Euganea, Via Pilastroni, 15, 35013 Cittadella (PD), Italy

**Keywords:** internal hernia, Roux-en-Y gastric bypass, bariatric surgery

## Abstract

We present the case of a patient with subocclusive crisis 1 year after undergoing Roux en-Y gastric bypass with an open approach. Laparoscopic exploration revealed a chylous ascites caused by an internal hernia through Petersen’s space; we found a technical error involving the creation of the jejuno-jejunal anastomosis below the alimentary limb, causing the inability to achieve the complete reduction of herniated small bowel. After the reduction of internal hernia, a new anastomosis was created between alimentary limb and bilio-pancreatic limb, bypassing the tract under the alimentary limb, and then Petersen’s space was closed.

## Introduction

**Figure 1:**
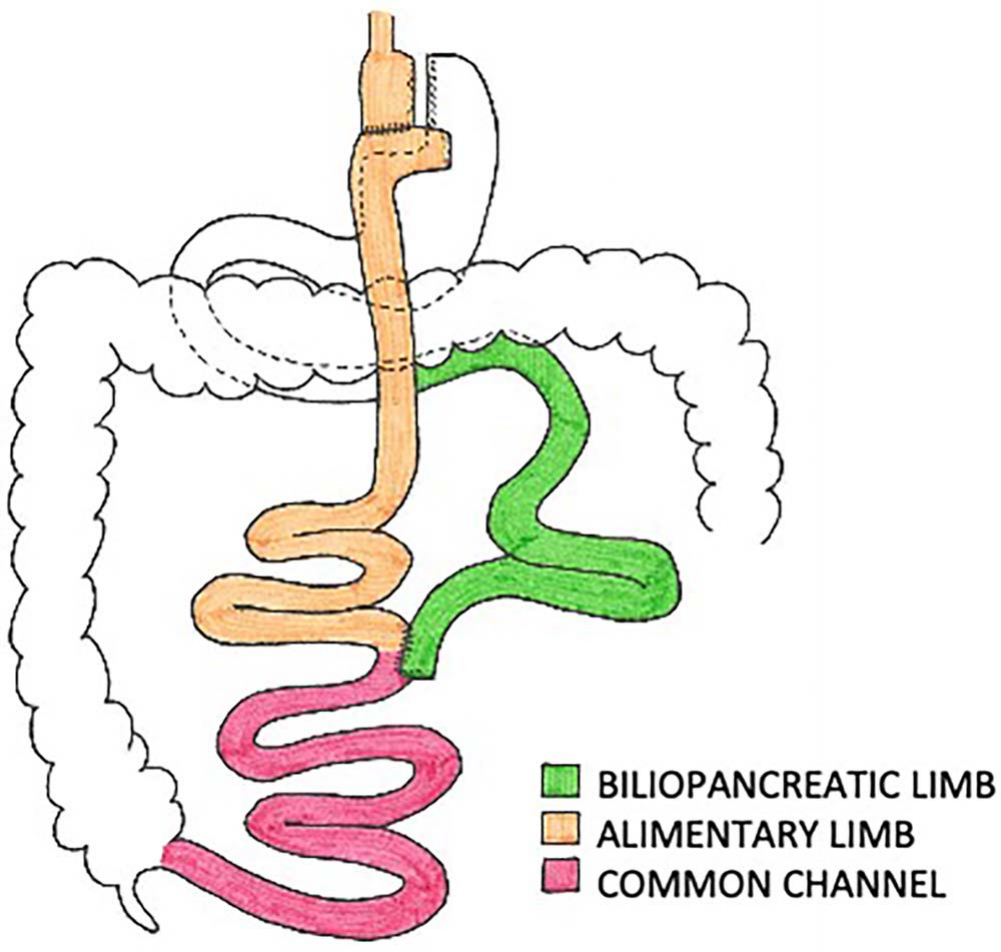
Standard RYGB.

Roux-en-Y gastric bypass (RYGB) (Fig. 1) is the second most commonly performed bariatric procedure worldwide, following sleeve gastrectomy [1].

The most common complication after RYGB is internal hernia (IH), with an incidence rate of 0.5%–11% [2]. Internal herniation is a serious complication caused by the slippage of small bowel loops through mesenteric defects created during surgery, the most common known as Petersen’s space hernia. Internal herniation commonly causes acute small bowel occlusion, with intense abdominal pain, nausea, and vomiting and it frequently occur between 1 and 2 years after surgery due to significant weight loss [3].

Diagnosis is often difficult and it is usually made combining clinical data, symptoms, and imaging. A computed tomography (CT) scan might show signs of small bowel occlusion with the “swirl sign” appearing as one of the most effective indicators of IH [4], but in some cases CT scan may be completely negative.

Urgent surgery is often needed to untwist the bowel and close the mesenteric defects.

## Case report

We present the case of a female patient, 47 years old who underwent laparotomic RYGB in an Eastern European Country on September 2024.

In the years prior to bariatric surgery, dieticians in our country had treated the patient. During all outpatient evaluations, the patient was deemed unsuitable for bariatric surgery due to her low body mass index (BMI). Patient was lost to follow-up on January 2024; at that time, before surgery, the patient was 96 kg, BMI 33.2.

**Figure 2:**
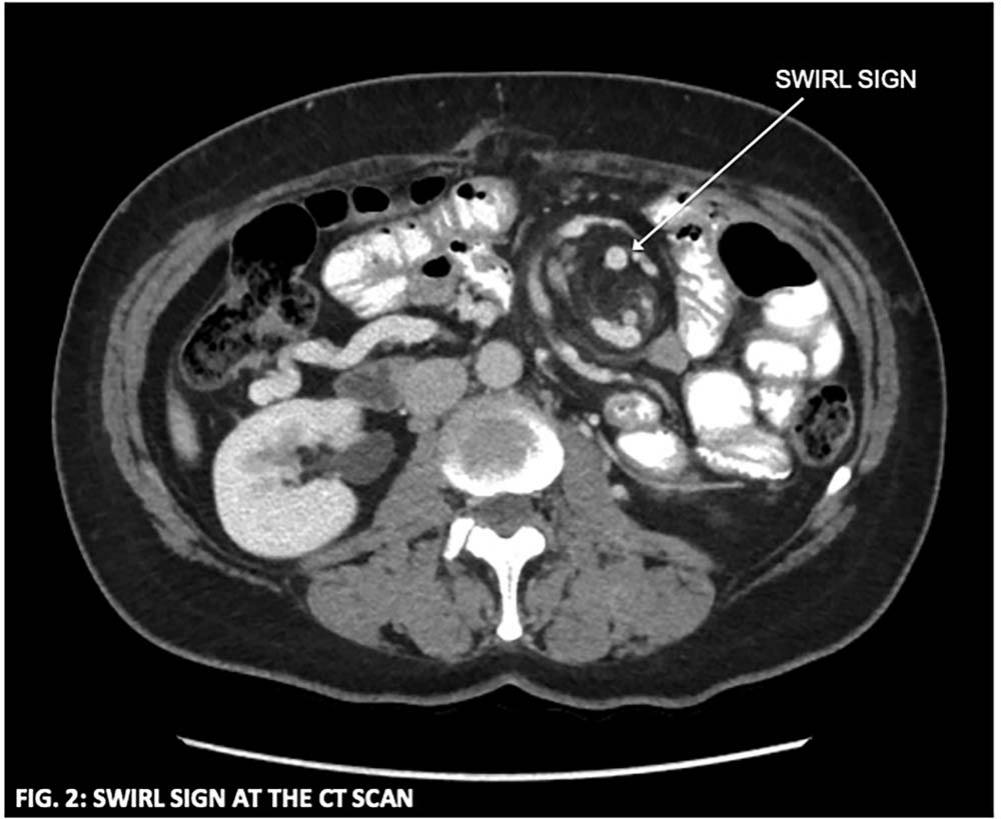
The “swirl sign” at CT scan.

About a year after surgery, on November 2025, the patient presented to our Emergency Department because of intermittent severe abdominal pain. All blood tests were normal, and the patient was evaluated by a bariatric surgeon due to her previous history. She underwent double-contrast (oral and intravenous) CT scan that showed the torsion of superior mesenteric vessels at their origin and the transposition of small bowel loops on the left side of abdomen under the transverse colon’s mesentery, suggestive for IH (likely a Petersen’s space hernia) (Fig. 2).

Since the clinical conditions and instrumental tests allowed it, the patient was discharged from the emergency department and was scheduled for an exploratory laparoscopy. After completing the preoperative assessments, patient underwent laparoscopic exploration on February 2026.

**Figure 3:**
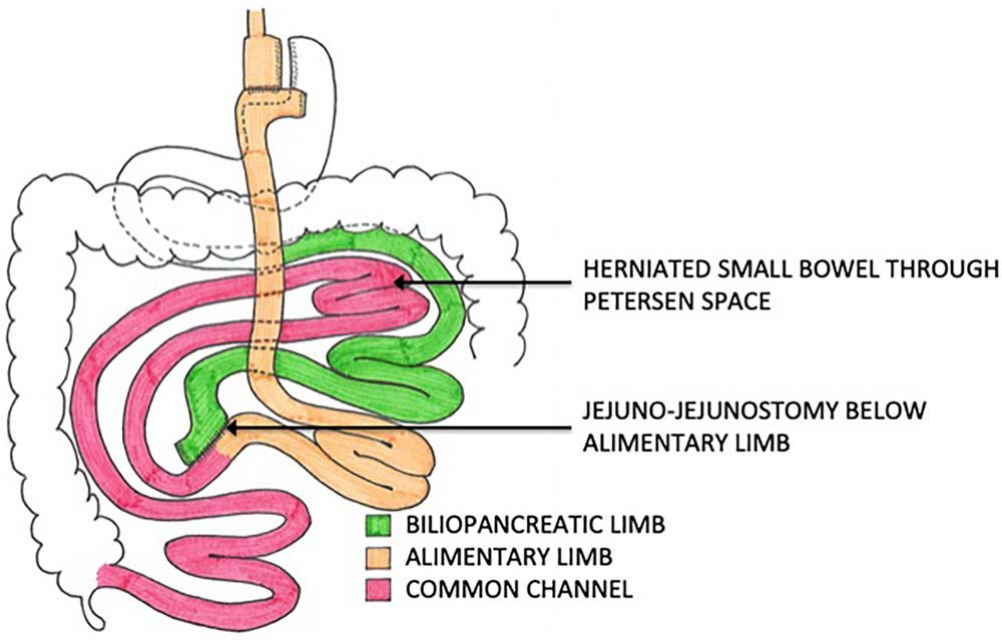
Initial condition at the time of laparoscopy.

**Figure 4:**
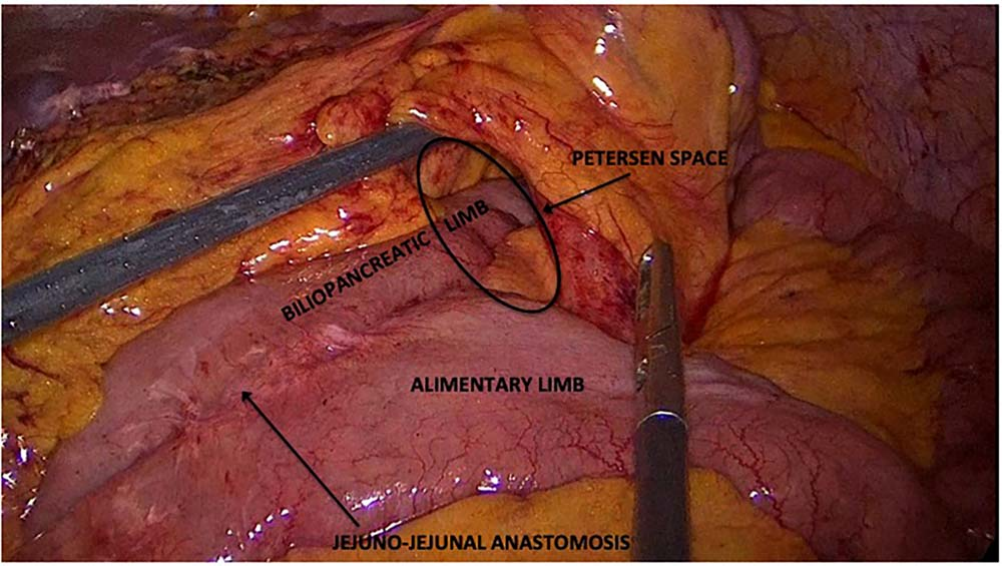
Internal hernia through Petersen’s space (the jejuno-jejunostomy is clearly visible under the alimentary limb).

Intraoperatively, extensive lysis of adhesions was required, due to multiple adhesions of the omentum to the abdominal wall, caused by previous laparotomy. A milky fluid was found in the pelvis and was aspirated for analysis that confirmed the suspicion of chylous ascites because of high lipid levels. The gastro-jejunal anastomosis was then identified. Below the alimentary limb a huge amount of small bowel was herniated and partially trapped through the Petersen’s space, which was left open in the previous operation. The IH was reduced and the herniated bowel was placed in its correct position. Then another problem emerged: the jejuno-jejunostomy was constructed with the bilio-pancreatic limb below the alimentary limb (Figs 3 and 4), making the complete reduction of this hernia truly impossible.

**Figure 5:**
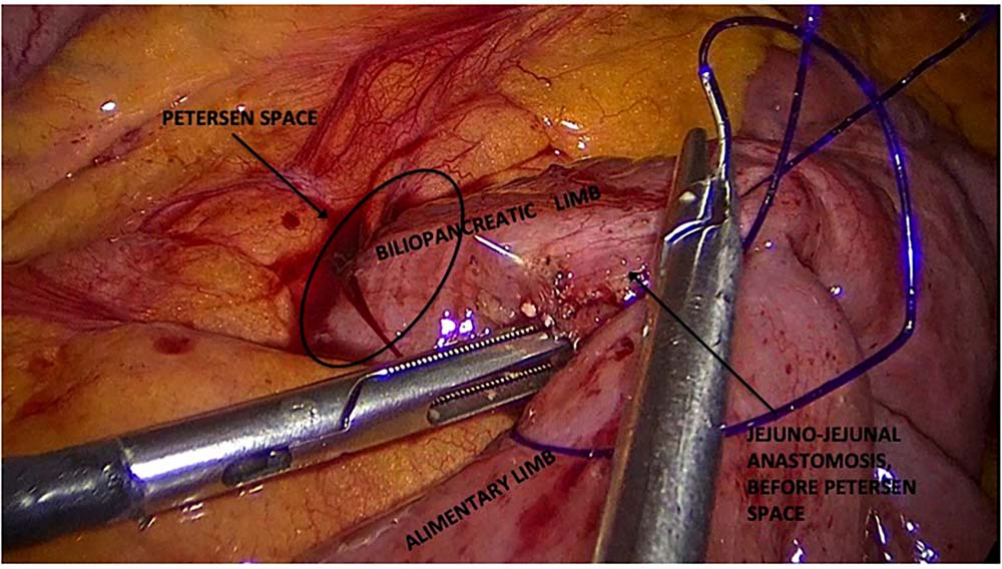
Creation of a new jejuno-jejunostomy.

**Figure 6:**
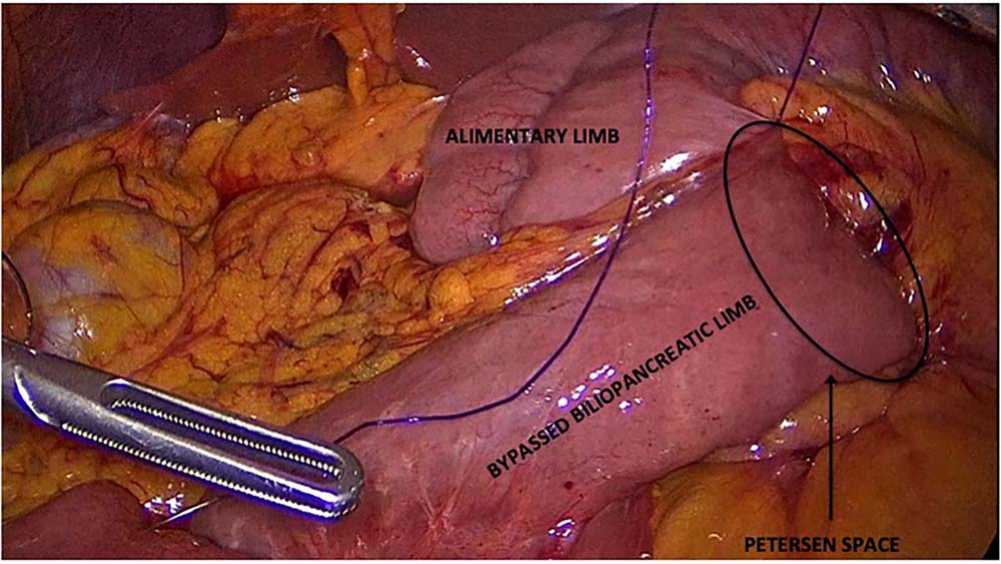
Closure of Petersen’s space.

**Figure 7:**
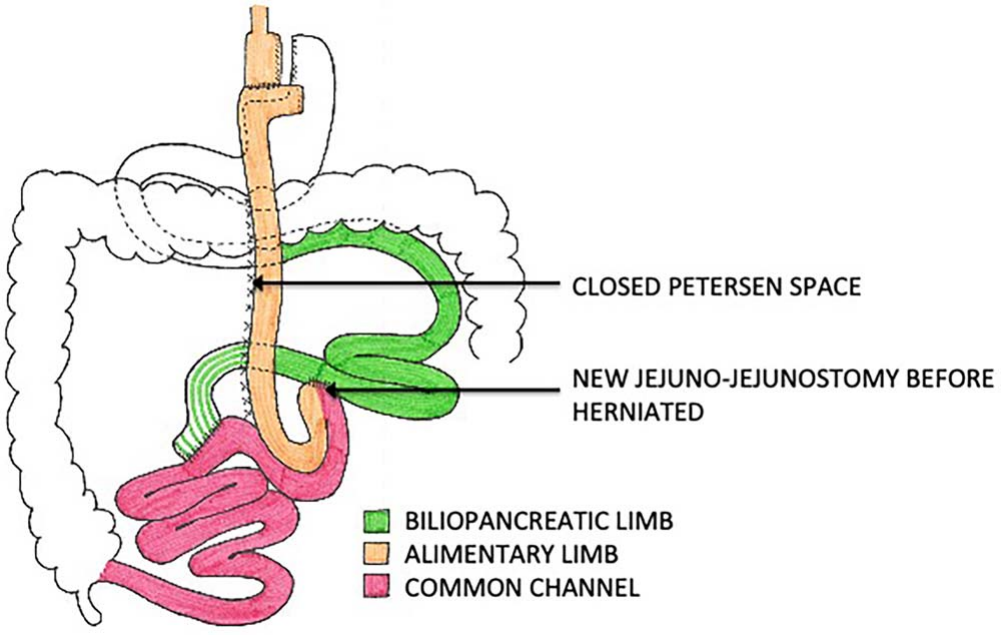
Final reconstruction.

To resolve this second problem it was decided to perform a second jejuno-jejunostomy before the passage of the biliopancreatic limb below the alimentary limb (Fig. 5). In this way, the herniated anastomosis was bypassed without the necessity of intestinal resection. Finally, Petersen’s space was closed with a running absorbable barbed suture (Fig. 6). Figure 7 represents the final reconstruction at the end of the operation.

All the operation was performed by laparoscopy, with an operative time of 225 min; there were no intra-operative complications.

Our patient was then treated with intravenous fluid therapy for 2 days, started soft oral diet on post-operative day 3 and was discharged on post-operative day 5. We did not observe any complication.

The subsequent follow-up was regular and the patient do not suffer from abdominal pain anymore.

## Discussion

Laparoscopic RYGB still represents the second most-performed bariatric operation worldwide, following sleeve gastrectomy, and it is performed over 200 000 times over year worldwide [1]. IH after RYGB occur when the intestine herniate through the mesenteric defects created by surgery. Two defects can be generated: the first is created with the mesenteric interruption in the location of the jejuno-jejunal anastomosis; the second, named Petersen’s space (first described by German surgeon Walther Petersen), originates with the antecolic reconstruction between the alimentary limb of the bypass and the transverse mesocolon [5]. In literature, an incidence rate of 0.5%–11% has been reported as well as a cumulative incidence of 4%–7% without closure of the mesenteric defects [4]. Another factor that contributes to IH formation is the rapid weight loss after surgery that can exacerbate mesenteric defects, facilitating bowel herniation [3]. Moreover the use of minimally invasive surgery, while favorably reducing adhesions, inadvertently increases the risk of IH formation due to greater bowel mobility [6].

Symptoms of IH are ambiguous and non-specific. The clinical presentation can vary from slight abdominal discomfort to intense pain. The severity of symptoms depends on the duration of the herniation, the reducibility of the hernia and the presence of strangulation or incarceration. Usually there is no rise in laboratory tests (such as WBC, renal function, liver function, or lactic acidosis) until there is infarction or perforation [7].

CT appear as the best imaging modality for the diagnosis of internal herniation after RYGB. Seven signs that can indicate an IH in a CT scan were studied by Lockhart *et al*. in 2007: swirl sign, superior mesenteric vein strangulation, engorged mesenteric vein and edema, enlarged lymphnode, ascites, mushroom sign, and hurricane eye [4]; another radiological sign is the right-sided location of the jejuno-jejunal anastomosis, at the right of the alimentary canal. The “swirl sign” is the rotation of the mesenteric vessels and fat and it is has been consistently associated with IH in the literature, with a sensitivity of 80% and a specificity of 90% [4]. However CT scan may still be inconclusive, particularly in cases with intermittent or self-reducing hernias [6].

The diagnosis of IH after RYGB is not so simple and given the risk of diffuse bowel incarceration, ischemia, and necrosis, a high level of suspicion is needed in patients with a history of RYGB presenting with abdominal complaints.

In this paper, we describe the case of a 47-year-old woman who had been followed by the dietitian of the local bariatric team for a long time. The patient had a BMI of 33, which was too low to be considered for bariatric surgery. Despite the Bariatric Team’s negative opinion, the patient independently decided to undergo bariatric surgery in her home country and she underwent a laparotomic RYGB. Approximately 1 year after surgery, she presented to the local emergency department complaining episodes of intense epigastric abdominal pain with nausea, vomiting, obstructed bowel movements, and gas, generally self-limiting with the lying position. All blood tests were normal. She underwent an abdominal CT scan, with endovenous and oral contrast-medium, showing the torsion of superior mesenteric vessels at their origin and the transposition of small bowel loops on the left side of abdomen under the mesocolic mesentery, suggesting an IH (likely a Petersen’s space hernia). In Fig. 2, the swirl sign is clearly visible, so as the transposition of a group of small bowel loops in the left side of abdomen.

Since patient’s conditions were permissive, an exploratory laparo-scopy was planned in the suspicion of a Petersen’s space hernia with intermittent episodes of blockage and subsequent reduction of the hernia. Interestingly, during laparoscopy we found a creamy-colored intra-abdominal accumulation of fluid (not visible in CT images) that was then found to contain high lipid levels, confirming the suspicion of chylous ascites. Chylous ascites is defined as a fluid collection with a triglyceride concentration of more than 110 mg/dl [8]. A cause of chylous ascites after RYGB is IH: it may form when the pressure from bowel obstruction, such in case of IH, obstructs normal lymphatic flow. It has been theorized that the presence of chylous ascites predicts salvageable bowel as the pressure to obstruct lymphatics is lower than that to obstruct vascular flow and cause bowel ischemia [9].

The success of non-surgical management of IH in the bariatric population is generally low as the mechanical nature of most obstructions post bypass surgery necessitates surgical correction [10]. In case of small bowel obstruction post laparoscopic RYGB, surgical option is the only viable option and nasogastric decompression is usually ineffective; generally the laparoscopic approach can be adopted in all patients, unless the patient is hemodynamically unstable. The recommended technique is the laparoscopic reduction of herniated small bowel loops.

Regarding the closure of mesenteric defects during RYGB, there are conflicting opinions. Most authors believe that closure of mesenteric defects is associated with a lower risk of IHs [6, 11]. However, Taselaar *et al*. observed in a large cohort of over 9000 patients who underwent RYGB, that the routine closure of mesenteric defects does not reduce the risk of clinically significant IH and that there was no reduction in re-operations for suspected IH; moreover the closure of mesenteric defects introduces a new complication, the risk of obstruction of the jejuno-jejunal anastomosis, known as kinking [12].

In our patient, we had to solve a further problem, linked to a technical error in the creation of the gastric bypass. The jejuno-jejunostomy was constructed with the bilio-pancreatic limb below the alimentary limb making the complete reduction of this hernia truly impossible without the resection of the anastomosis and the creation of a new one. So we decided to perform a second jejuno-jejunostomy before the passage of the bilio-pancreatic limb below the alimentary limb (Fig. 5). In this way, the herniated anastomosis was bypassed without the necessity of intestinal resection. Finally, Petersen’s space was closed with a running barbed suture (Fig. 6).

For the prevention of IH after RYGB, Hwang *et al*. suggested the antecolic position of the alimentary limb that significantly decreased the risk of small bowel occlusion compared to the retrocolic position [13].

Another technique for the same purpose is the “Double Loop” gastric bypass technique, first described by Tacchino *et al*. in 2010 [14]; this technique avoids the opening of mesentery during the construction of the Roux limb: the transection of the small bowel without opening the mesentery fixes the gastro-jejunal and the jejuno-jejunal anastomoses close to each other in the supramesocolic compartment and in this way the mesentery integrity is preserved. Rebecchi *et al*. in 2021 [15] diagnosed no IH after the application of the “Double Loop” gastric bypass technique in a large group of patients with a long follow-up.

## Conclusions

IH after RYGB is a serious complication. It can manifest with a variable pattern, from subocclusive crisis to acute obstruction with intestinal ischemia and necrosis. Diagnosis is difficult and requires a high level of suspicion and the integration of clinical and radiological data. There are several technical measures to reduce the risk of IH. Patients must be monitored over time to identify this potentially lethal complication early.

This case is emblematic because it reports both a very rare type of IH, partially caused by a wrong construction of the biliopancreatic limb below the alimentary limb, and an interesting trick to resolve it, bypassing it with a new anastomosis without any intestinal resection.

## Data Availability

No data are available because this study did not generate any datasets.
